# CRISPR/Cas9 nickase mediated signal amplification integrating with the trans-cleavage activity of Cas12a for highly selective and sensitive detection of single base mutations

**DOI:** 10.1186/s40779-024-00530-x

**Published:** 2024-04-23

**Authors:** Xiao-Wen Fan, Zi-Fan Gao, Dong-Dong Ling, De-Hui Wang, Ying Cui, Hui-Qun Du, Chun-Lin Li, Xing Zhou

**Affiliations:** 1 Jinling Clinical Medical College, Nanjing University of Chinese Medicine, Nanjing, 210002 China; 2 Department of Orthopedics, Affiliated Hospital of Medical School, Jinling Hospital, Nanjing University, Nanjing, 210002 Jiangsu China; 3 Department of Health Medicine, the Eighth Medical Center of PLA General Hospital, Beijing, 100093 China

**Keywords:** Cas9 nickase, CRISPR-Cas12a, Polymerase, Single base mutations

Dear Editor,

Mutations in genomic sequences exhibit a strong correlation with various pathological processes of cancers [1]. Currently, the next-generation sequencing technique [2] and polymerase chain reaction (PCR) were the established benchmarks for analyzing DNA mutations. However, the two methods necessitate intricate experimental preparation, costly instrumentation, and skilled personnel, making them challenging for rapid mutations analysis. More importantly, these methods lack adequate accuracy for one base mutations analysis [3]. Therefore, the development of a reliable and exceptionally sensitive mutation analysis approach holds immense importance in cancer diagnosis and treatment.

Clustered regularly interspaced short palindromic repeats (CRISPR)/CRISPR-associated (CRISPR/Cas) systems have facilitated the development of nucleic acid sensing techniques with improved selectivity to even one base mismatch. Among them, Cas9 nickase, a variant of Cas9 protein has attracted abundant attention due to its unique capability of generating nicks in double-stranded DNA (dsDNA) but lacking the ability to cleave it [4]. Various platforms have been established utilizing the Cas9 nickase for identification of specific genomic sites [5, 6], but lack enough sensitivity for low abundant mutation detection.

In this study, we provide a unique diagnostic platform that combines a Cas9 nickase and Cas12a to ensure single base mutations analysis with an improved sensitivity. As shown in Fig. 1a, the Cas9 nickase/CRISPR RNA (crRNA) complex has unique affinity for the mutated gene sequences, resulting in a nicking site where the subsequent polymerase/endonuclease assisted signal recycling can occur. This nick serves as a location for the replacement probe (“Rep” probe). The “Rep” probe and mutated fragment were extended at their 3’ terminus with the aid of Klenow fragment (KF), transcribing the “recognizing site” on the “Rep”. Subsequently, the endonuclease identifies the recognizing site, leading to the formation of a single-strand break. Through the collaborative efforts of polymerase and endonuclease, numerous “Rec” chains were generated. Afterwards, the Cas12a/crRNA complex identifies the released “Rec” chains, thereby inducing the activation of Cas12a’s trans-cleavage activity. A single active Cas12a can cut thousands of “Reporter” probes, significantly enhancing the reaction intensity. The approach’s performance was validated through detecting a mutation (KRAS-G12D) in the *KRAS* gene (Additional file 1: Materials and methods, Table S1).

**Fig. 1:**
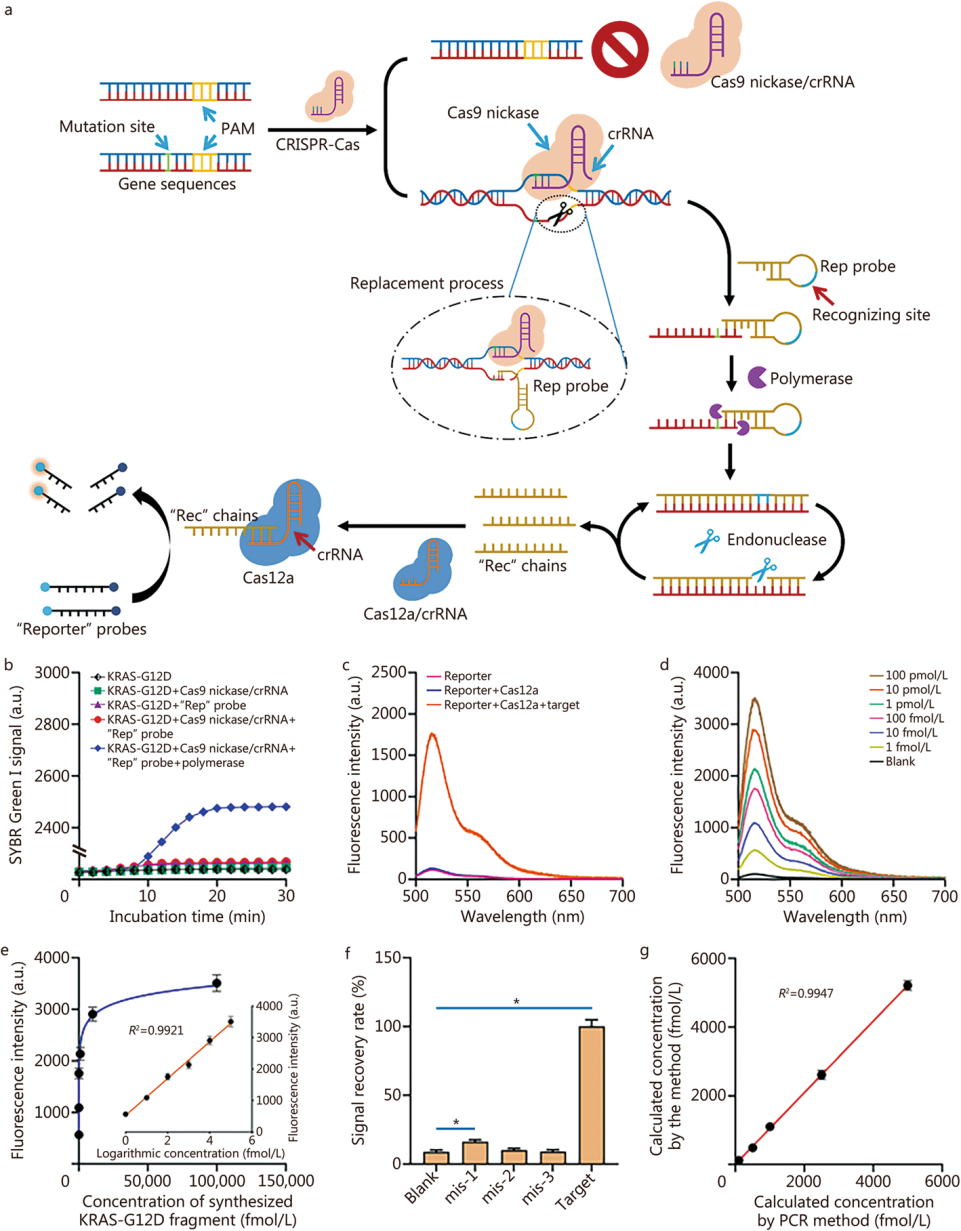
Construction of the accurate and sensitive mutation analysis platform. **a** The working mechanism of the method for identifying single base mutation. **b** SYBR Green I signal during chain extension process. **c** Fluorescence spectrum of the “Reporter” in the presence or absence of target sequence. **d** Fluorescence spectrum of the approach when detecting different concentrations of synthesized KRAS-G12D fragment. **e** Correlation between the recorded fluorescence intensities and the concentration of synthesized KRAS-G12D fragment. **f** Signal recovery rate of the approach for different mutated sequences detection. **g** Correlation between the calculated concentration by the method and by the polymerase chain reaction (PCR). ^*^P < 0.05. CRISPR-Cas9 clustered regularly interspaced short palindromic repeats (CRISPR)/CRISPR-associated 9, crRNA CRISPR RNA, PAM protospacer adjacent motif

The differences in cleavage capability of Cas9 and Cas9 nickase were analyzed through fluorescent assays. The results demonstrated that Cas9 nickase/crRNA could only generate a nick in single-stranded DNA (ssDNA; Additional file 1: Fig. S1). The separation fluorescence experiment verified the detection and binding of mutated fragment by “Rep” probes via incisions (Additional file 1: Fig. S2). The outcome depicted in Fig. 1b illustrates the temporal variations in SYBR Green I signals, and a comparison was made between the intensities observed at equilibrium. The fluorescence signal of SYBR Green I exhibited a consistently low level only Cas9 nickase/crRNA complex existed. A significantly increased SYBR Green I signal was observed only when Cas9 nickase/crRNA, KF, and “Rep” co-existed, implying their crucial roles in initiating chain displacement and extension. The trans-cleavage activity of the CRISPR-Cas12a system is crucial in facilitating second signal amplification. As depicted in Fig. 1c, the fluorescence assay demonstrated the successful cleavage of the “Reporter” probes by Cas12a/crRNA, as indicated by the orange line. After optimizing the experimental parameters, we assessed the sensitivity of the approach through detecting manufactured KRAS-G12D fragments at varying doses under the experimental parameters of 30 nmol/L of Cas12, 50 nmol/L of Cas9 nickase, 0.2 U/μl of KF, and 500 nmol/L of “Reporter” probe (Additional file 1: Fig. S3). As shown in Fig. 1d, the fluorescence intensity demonstrated a positive correlation with the escalating concentration of target (1 fmol/L to 100 pmol/L). The data indicated a strong linear association (Y = 586.5 × lgC + 527.3, *R*^2^ = 0.9921) as illustrated in Fig. 1e. The method’s low limit of detection (LOD) was determined to be 0.24 fmol/L using the 3σ method. Moreover, the selectivity was demonstrated through the differences between F/F_0_ (F, fluorescence intensities for target detection; F_0_, fluorescence intensity for blank sample detection) of the approach for different sequences detection in Additional file 1: Fig. S4 and Fig. 1f. The findings indicated that the suggested method achieves signal recovery rates of 16.32%, 10.21% and 9.12% for the sequences containing one base (mis-1), two base (mis-2) or three base mismatches (mis-3), respectively. While the signal recovery for mis-1 sequence is larger compared to blank sample, it remains very low and can be easily differentiated from the signal associated with target identification (*P* < 0.05, Fig. 1f). The result in Additional file 1: Fig. S5a showed no significant differences between the fluorescence intensities for 10 fmol/L and 1 pmol/L KRAS-G12D detection with different concentrations of mtDNA, verifying wild-type DNA present in the sensing system had minimal impact on the detection of KRAS-G12D, and possessed a high stability for mutation analysis (Additional file 1: Fig. S5b). The result in Fig. 1g showed a strong positive connection between the target concentration by the developed method and PCR. Additional file 1: Fig. S6 showed both the method and PCR can report the KRAS-G12D mutation positive samples with a high consistency. The above results strongly indicate the potential clinical applications of the proposed platform. The advantages of this platform over other reported platforms are compared in Additional file 1: Table S2.

In conclusion, this paper study introduces a new platform for analyzing single base mutation using Cas9 nickase and incorporating the CRISPR-Cas12a system for signal generation. The diagnostic tool exhibits a low LOD of 0.24 fmol/L, and displays specificity in recognizing single base mutation accurately. The platform’s sensitivity closely matches that of PCR, a traditional and widely-used method for nucleic acid analysis, making it suitable for practical samples detection. This technology has the potential to efficiently detect complex samples with high sensitivity, offering promising applications in clinical diagnostics and patient care.

### Supplementary Information

**Additional file 1: Materials and methods. Fig. S1** Fluorescence spectrum of the FAM labeled mutated fragment (mt-F) when mixed with CRISPR-Cas9 or Cas9 nickase/crRNA. **Fig. S2** Recognition of the nicking site by the “Rep” probe. **Fig. S3** Optimization of experimental parameters. **Fig. S4** Selectivity of the approach to mismatched sequences. **Fig. S5** Stability of the proposed approach. **Fig. S6** Heat map of the KRAS-G12D mutation positive samples reported by the proposed CRISPR/Cas9 nickase mediated signal amplification-based method and PCR.** Table S1** The sequences of oligonucleotide in this study. **Table S2** A brief comparison of the approach with former ones.

## Data Availability

The data and materials used to support the findings of this study are available from the corresponding authors upon request.

## Abbreviations

CRISPR: Clustered regularly interspaced short palindromic repeats
PCR: Polymerase chain reaction
KF: Klenow fragment
ssDNA: Single-stranded DNA
dsDNA: Double-stranded DNA
LOD: Limit of detection
sgRNA: Small guide RNA
