# Supplementary material for: CRISPR/Cas9 nickase mediated signal amplification integrating with the trans-cleavage activity of Cas12a for highly selective and sensitive detection of single base mutations
Source: Mil Med Res. 2024 Apr 23;11:25. doi: 10.1186/s40779-024-00530-x (PMC11036582; doi:10.1186/s40779-024-00530-x)
Supplement: Supplementary file 1 — Additional file 1: Materials and methods. Fig. S1 Fluorescence spectrum of the FAM labeled mutated fragment (mt-F) when mixed with CRISPR-Cas9 or Cas9 nickase/crRNA. Fig. S2 Recognition of the nicking site by the “Rep” probe. Fig. S3 Optimization of experimental parameters. Fig. S4 Selectivity of the approach to mismatched sequences. Fig. S5 Stability of the proposed approach. Fig. S6 Heat map of the KRAS-G12D mutation positive samples reported by the proposed CRISPR/Cas9 nickase mediated signal amplification-based method and PCR. Table S1 The sequences of oligonucleotide in this study. Table S2 A brief comparison of the approach with former ones. [file 40779_2024_530_MOESM1_ESM.pdf]

Materials and methods

Materials and reagents

The oligonucleotide employed in this research were synthesized and purified by Shanghai Sangon Biotech. Co. Ltd. (Shanghai, China). The detail of the oligonucleotides is listed in **Additional file 1: Table S1**. Cas9 nickase, EnGen® Lba Cas12a, Klenow fragment (KF), and nicking endonuclease Nb.BbvCI were purchased from New England Biolabs (Beijing, China). Deoxyribonucleoside 5'-triphosphate mixture (dNTPs) was obtained from Tiangen Biotech. Co. Ltd. (Beijing, China). The HEK293T cells utilized in this study were acquired from the Cell Bank, Chinese Academy of Sciences (Shanghai, China).

Feasibility of the target recognition of Cas9 nickase

The experiments involved the use of Cas9 nickase-mediated DNA cleavage assays, which were conducted in a reaction volume of 20 μl. Initially, the combination consisting of 100 nmol/L Cas9 nickase protein, 100 nmol/L crRNA, and 1× Tris buffer (composed of 40 mmol/L Tris-HCl, 60 mmol/L NaCl, and 6 mmol/L $MgCl_2$, with a pH of 7.4) was subjected to incubation at room temperature for 5 min, resulting in the formation of the Cas9/crRNA complex. Subsequently, the designated DNA sequences (KRAS-G12D) were introduced into the reaction system, resulting in a total volume of 20 μl. The reaction mixture was then subjected to incubation at either room temperature or 37 °C for a duration ranging from 10 to 60 min, facilitating the process of cleavage.

Analytical performance

Three steps were included for mutation analysis. Firstly, 50 nmol/L Cas9 nickase (5 μl), 50 nmol/L crRNA (5 μl), 5 μl of the "Rep" probe, and different concentrations of KRAS-G12D double-stranded DNA (dsDNA) sequences (5 μl) were mixed in a tube containing 5 μl of 1× PBS buffer solution. The mixture is incubated at room temperature for 30 min to mediate the target cleavage and chain replacement. Afterwards, 2 μl of KF, 2 μl of the nicking endonuclease Nb.BbvCI, 1 μl of the 0.33 mmol/L dNTP, and 6 μl of the 1× Tris buffer were added to the mixture. The mixture was then incubated at room temperature for 30 min to produce abundant "Rec" chains. Afterwards, 30 nmol/L Cas12a, 30 nmol/L small guide RNA (sgRNA; Cas12a), and 500 nmol/L "Reporter" probes were added to the mixture. The reaction system was incubated at room temperature for 30 min and the fluorescence signals were recorded.

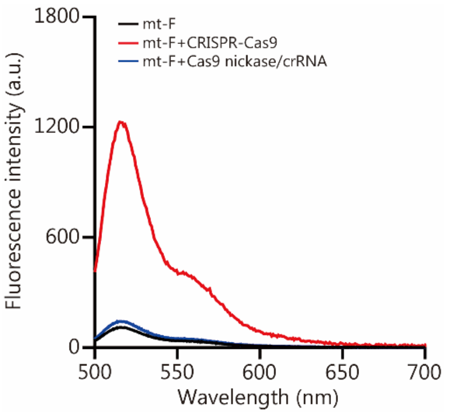

Fig. S1 Fluorescence spectrum of the FAM labeled mutated fragment (mt-F) when mixed with CRISPR-Cas9 or Cas9 nickase/crRNA. FAM carboxyfluorescein, CRISPR-Cas9 clustered regularly interspaced short palindromic repeats (CRISPR)/CRISPR-associated 9, crRNA CRISPR RNA

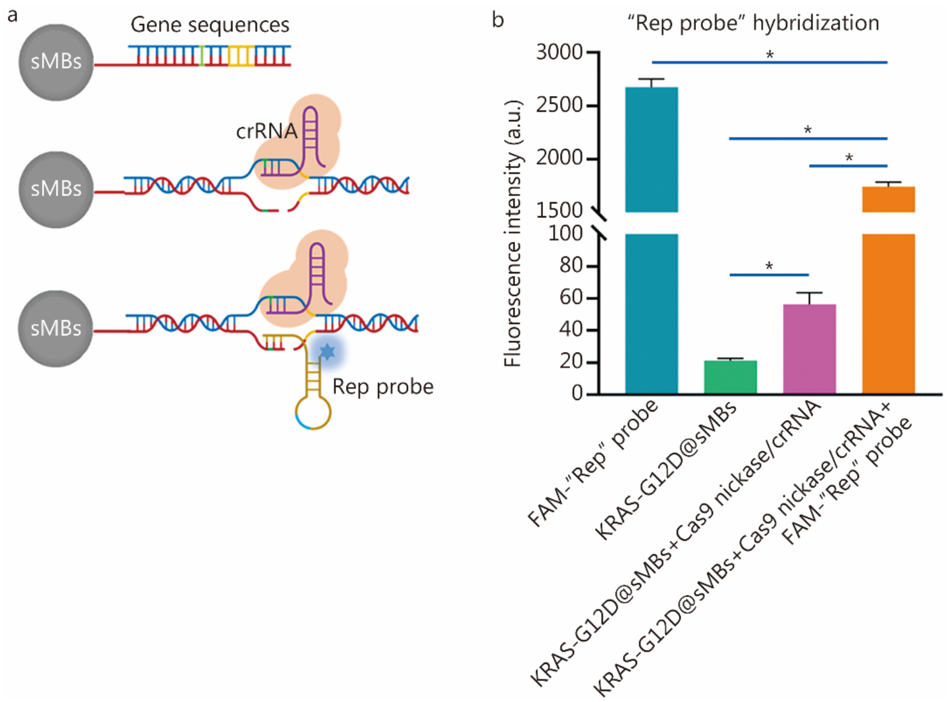

Fig. S2 Recognition of the nicking site by the "Rep" probe. **a** Illustration of the fluorescent assay to test the recognizing capability of the FAM labeled "Rep" probe. **b** Fluorescence intensities of the FAM labeled "Rep" probe during the hybridization process. KRAS Kirsten rat sarcoma viral oncogene homolog, FAM carboxyfluorescein, sMBs streptavidin magnetic beads, crRNA clustered regularly interspaced short palindromic repeats RNA. $^{*}P < 0.05$

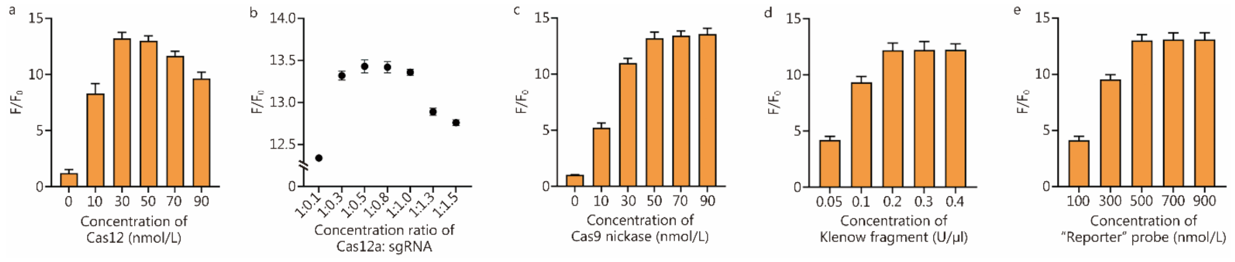

Fig. S3 Optimization of experimental parameters. Fluorescence ratio (F/F0) of the approach with different concentrations of Cas12a (**a**), concentration ratio of Cas12a and small guide RNA (sgRNA; **b**), and concentration of Cas9 nickase (**c**), Klenow fragment (3'→ 5') (**d**), "Reporter" probe (**e**)

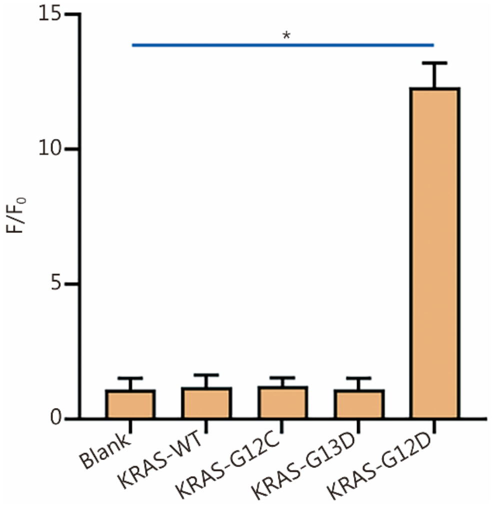

Fig. S4 Selectivity of the approach to mismatched sequences. F/F_0 of the approach for different sequences detection. KRAS Kirsten rat sarcoma viral oncogene homolog

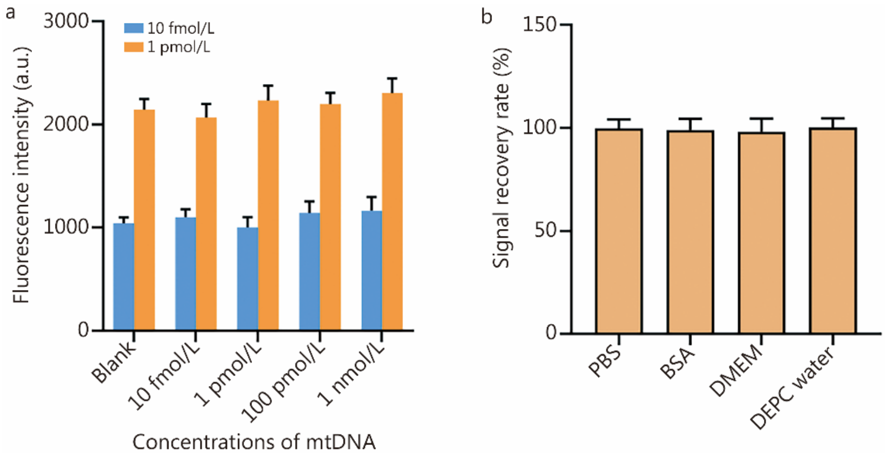

Fig. S5 Stability of the proposed approach. **a** Fluorescence intensities of the approach when detecting 10 fmol/L and 1 pmol/L synthesized KRAS-G12D fragment in solution containing different concentrations of mtDNA sequences. **b** Signal recovery rate of the approach when detecting target in complex matrix. mtDNA mutated DNA, PBS phosphate buffer solution, BSA bovine serum albumin, DMEM dulbecco's modified eagle medium, DEPC diethyl pyrocarbonate

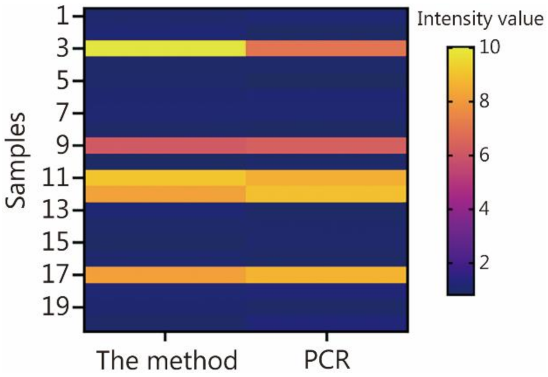

Fig. S6 Heat map of the KRAS-G12D mutation positive samples reported by the proposed CRISPR/Cas9 nickase mediated signal amplification-based method and PCR. KRAS Kirsten rat sarcoma viral oncogene homolog, CRISPR-Cas9 clustered regularly interspaced short palindromic repeats (CRISPR)/CRISPR-associated 9, PCR polymerase chain reaction

Table S1 The sequences of oligonucleotide in this study

**Name**	**Sequences (5' – 3')**
KRAS-F	TGCTGAAAATGACTGAATATAAACTTGTGGTAG
KRAS-R	TATTCGTCCACAAAATGATTCTGAATTAGCTGTATCGTCAAGCCACTCTTGCCTACG
sgRNA	TCTAATACGACTCACTATAGGGGTAGTTGGAGCTGATGGCGTGTTTTAGAGCTAGA
Reporter probe	AATTTTATTTAA (5' 6-FAM; 3' BHQ1)

KRAS Kirsten rat sarcoma viral oncogene homolog, *sgRNA* small guide RNA, *FAM* carboxyfluorescein, *BHQ1* black hole quencher 1

Table S2 A brief comparison of the approach with former ones

Item	Mechanism	Sensitivity	Mutations	Enzymes	Advantages and disadvantages	Ref.
The method	Cas9 nickase for recognition; Cas12a/crRNA for signal amplification	0.24 fmol/L	Single base mutation	Cas9; Cas12a; DNA polymerase	Advantages: high sensitivity; the dual check by the Cas9 and Cas12a protein endows the method a high selectivity; Disadvantages: require multiple enzymes	
Cas9 system	CRISPR/Cas9-mediated proximity ligation assay	Not mention	Single-nucleotide variation	Cas9; T4 DNA ligase; phi29 polymerase	Advantages: high selectivity; Disadvantages: the proximity ligation process may induce wrong amplification, and thus interfering the selectivity of the method	[1]
eiCRISPR	DNAzyme + CRISPR-Cas9	Nanomole level	Gene editing	Cas9	Advantages: the method can be applied for gene editing; Disadvantages: low sensitivity	[2]
Triple signal amplification platform	CRISPR-Cas9 + RCA	2 fmol/L	Single base mutation	Cas9; T4 DNA ligase; phi29 polymerase	Advantages: high selectivity; Disadvantages: multiple enzymes; the RCA process may arise wrong amplifications	[3]

CRISPR-Cas9 clustered regularly interspaced short palindromic repeats (CRISPR)/CRISPR-associated 9, *RCA* rolling circle amplification, *crRNA* clustered regularly interspaced short palindromic repeats RNA, *eiCRISPR* enzyme-inducible clustered regularly interspaced short palindromic repeats

References

1. Zhang K, Deng R, Teng X, Li Y, Sun Y, Ren X, et al. Direct visualization of single-nucleotide variation in mtDNA using a CRISPR/Cas9-mediated proximity ligation assay. J Am Chem Soc. 2018;140(36):11293-301.

2. Cai W, Liu J, Chen X, Mao L, Wang M. Orthogonal chemical activation of enzyme-inducible CRISPR/Cas9 for cell-selective genome editing. J Am Chem Soc. 2022;144(48):22272-80.

3. Zhou M, Wang H, Li C, Yan C, Qin P, Huang L. CRISPR/Cas9 mediated triple signal amplification platform for high selective and sensitive detection of single base mutations. Anal Chim Acta. 2022;1230:340421.
